# Supplementary material for: Propensity of Tagetes erecta L., a Medicinal Plant Commonly Used in Diabetes Management, to Accumulate Perfluoroalkyl Substances
Source: Toxics. 2019 Mar 25;7(1):18. doi: 10.3390/toxics7010018 (PMC6468628; doi:10.3390/toxics7010018)
Supplement: Supplementary file 1 [file toxics-07-00018-s001.pdf]

Supplementary Materials: Propensity of *Tagetes erecta* L., a Medicinal Plant Commonly Used in Diabetes Management, to Accumulate Perfluoroalkyl Substances

John Baptist Nzukizi Mudumbi *, Adegbenro Peter Daso, Okechukwu Jonathan Okonkwo, Seteno Karabo Obed Ntwampe, Tandi E. Matsha, Lukhanyo Mekuto, Elie Fereche Itoba-Tombo, Adewole T. Adetunji and Linda L. Sibali

Table S1. Selected medicinal plants under possible threats by PFASs in South Africa [1].

Plant Species (Family)	Common or Vernacular Names	Compartments Used	References
Tagetes erecta (*Asteraceae*)	African marigold (Eng.)	Leaves and roots	This study, [2–7]
Sutherlandia frutescens (*Fabaceae*)	Cancer bush (Eng.)	Leaves, and often whole plant	[8–10]
Moringa oleifera (*Moringaceae*)	Makgonatˇsohle (Sipedi), drumstick tree (Eng.)	Seeds and leaves	[11]
Artemisia afra (*Asteraceae*)	African Wormwood (Eng.)	Leaves and roots	[8,12–14]
Cannabis sativa L. (*Cannabaceae*)	Dagga (Afr.)	Leaves	[15]
Aloe ferox Mill. (*Asphodelaceae*)	Cape Aloe or bitter Aloe (Eng.)	Leaves	[10,16–18]
Pelargonium sidoides (*Geraniaceae*)	Umckaloabo (Zulu)	Tubers and roots	[10]
Hypoxis hemerocallidea (*Hypoxidaceae*)	Star flower, yellow star, African potato (Eng.); Inkomfe (Zulu); Sterblom and Gifbol (Afr.)	Roots	[10,18–20]
Sclerocarya birrea (*Anacardiaceae*)	Hochst. subsp. caffra, marula, tree of life	Stem	[10,21]
Herichrysum nudifolium L. (*Asteraceae*)	Hottentot's tea (Eng.); Hottentotstee (Afr.); icholocholo (Xhosa, Zulu)	Leaves and roots	[12,14]
Herichrysum petiolare H & B.L (*Asteraceae*)	Everlasting (Eng.); Kooigoed (Afr.); Imphepho (Xhosa)	Whole plant	[12,14]
Leonotis leonurus L. (*Lamiaceae*)	Wild dagga or Lion's ear (Eng.); Wildedagga (Afr.); Imvovo (Xhosa)	Leaves, flowers	[13,14]
Momordica balsamina L. (*Cucurbitaceae*)	Balsam pear (Eng.); Laloentjie (Afr.); Nkaka (Thonga) Intshungu (Zulu)	Stem, flowers	[14,15]
Momordica foetida Schumach (*Cucurbitaceae*)	Wild cucumber (Eng.)	Leaves, and often whole plant	[14,15,22,23]
Psidium guajava L. (*Myrtaceae*)	Common guava, yellow guava, lemon guava (Eng.)	Leaves, roots, whole plant	[14,15,24]
Sclerocarya birrea Hochst (*Anacardiaceae*)	Marula (Eng.); Mufula (Venda)	Stem, bark, roots	[14,15]
Vinca major L. (*Apocynaceae*)	Bigleaf periwinkle (Eng.)	Leaves, roots, stem	[14,15]
Vernonia oligocephala Sch. Bip. (*Asteraceae*)	Bicoloured-leaved Vernonia (Eng.); Groenamarabossie (Afr.); Ihlambihloshane (Zulu)	Leaves, twigs, roots	[12,14]
Catha edulis Forrsk. Ex Endl. (*Celastraceae*)	Arabian tea, Abyssinian tea, Bushman's tea (Eng.)	Leaves, stems, roots	[14,15]
Brachylaena discolor DC. (*Asteraceae*)	Coast silver oak (Eng.) ; Kusvaalbos (Afr.); Phahla (Zulu and Xhosa)	Leaves, roots, stem	[12,14,15]
Eriocephalus punctulatus (*Asteraceae*)	Roosmaryn or Kapokbos (Afr.); wild rosemary (Eng.)	Leaves	[18,25–27]

Afr. = Afrikaans; Eng. = English.

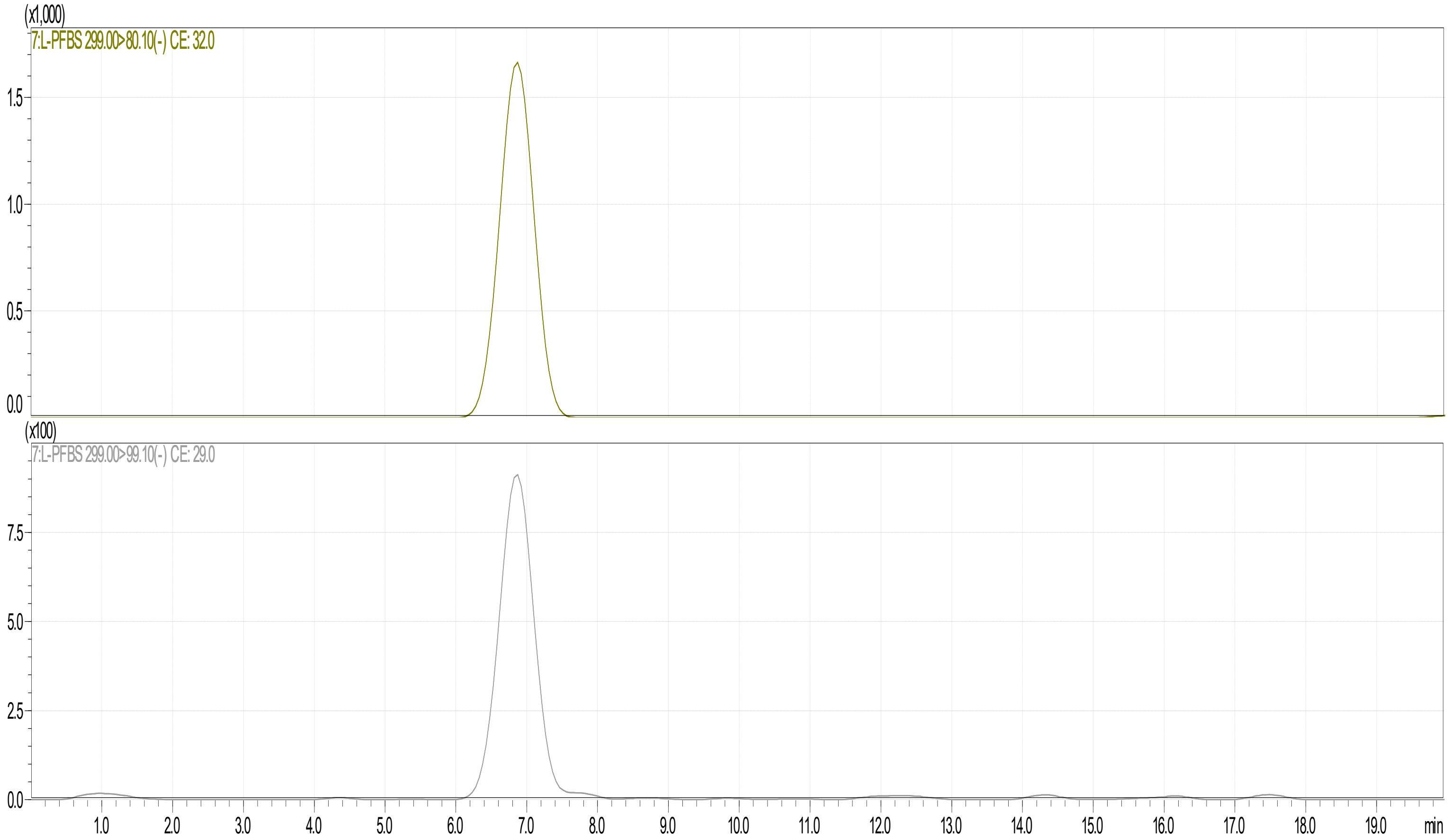
(x1,000)
7:L-PFBS 299.00>80.10(-) CE: 32.0
1.5
1.0
0.5
0.0
(x100)
7:L-PFBS 299.00>99.10(-) CE: 29.0
7.5
5.0
2.5
0.0
1.0
2.0
3.0
4.0
5.0
6.0
7.0
8.0
9.0
10.0
11.0
12.0
13.0
14.0
15.0
16.0
17.0
18.0
19.0
min

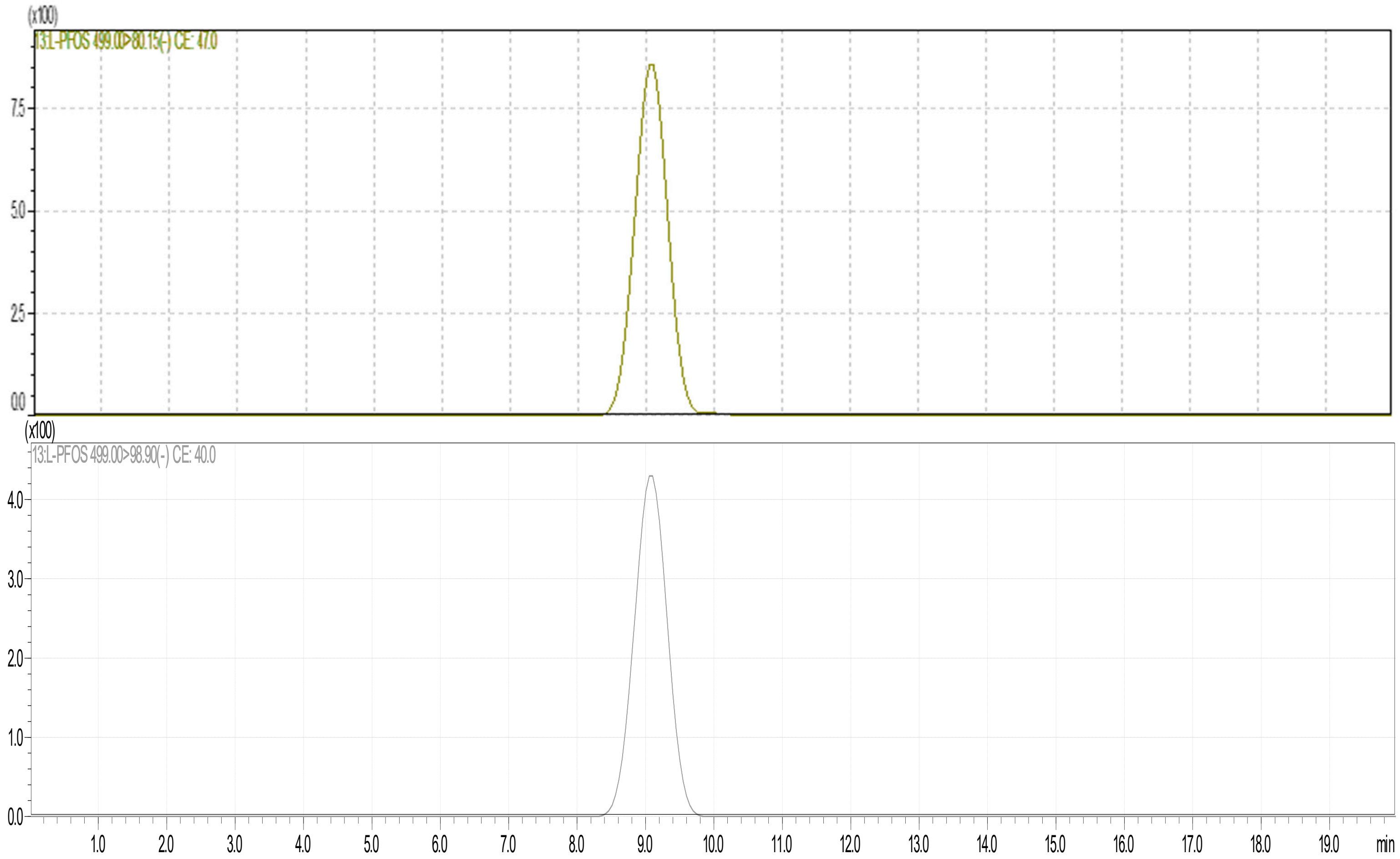
(x100)
13:L-PFOS 499.00>80.15(-) CE: 47.0
7.5
5.0
2.5
0.0
(x100)
13:L-PFOS 499.00>98.90(-) CE: 40.0
4.0
3.0
2.0
1.0
0.0
1.0
2.0
3.0
4.0
5.0
6.0
7.0
8.0
9.0
10.0
11.0
12.0
13.0
14.0
15.0
16.0
17.0
18.0
19.0
min

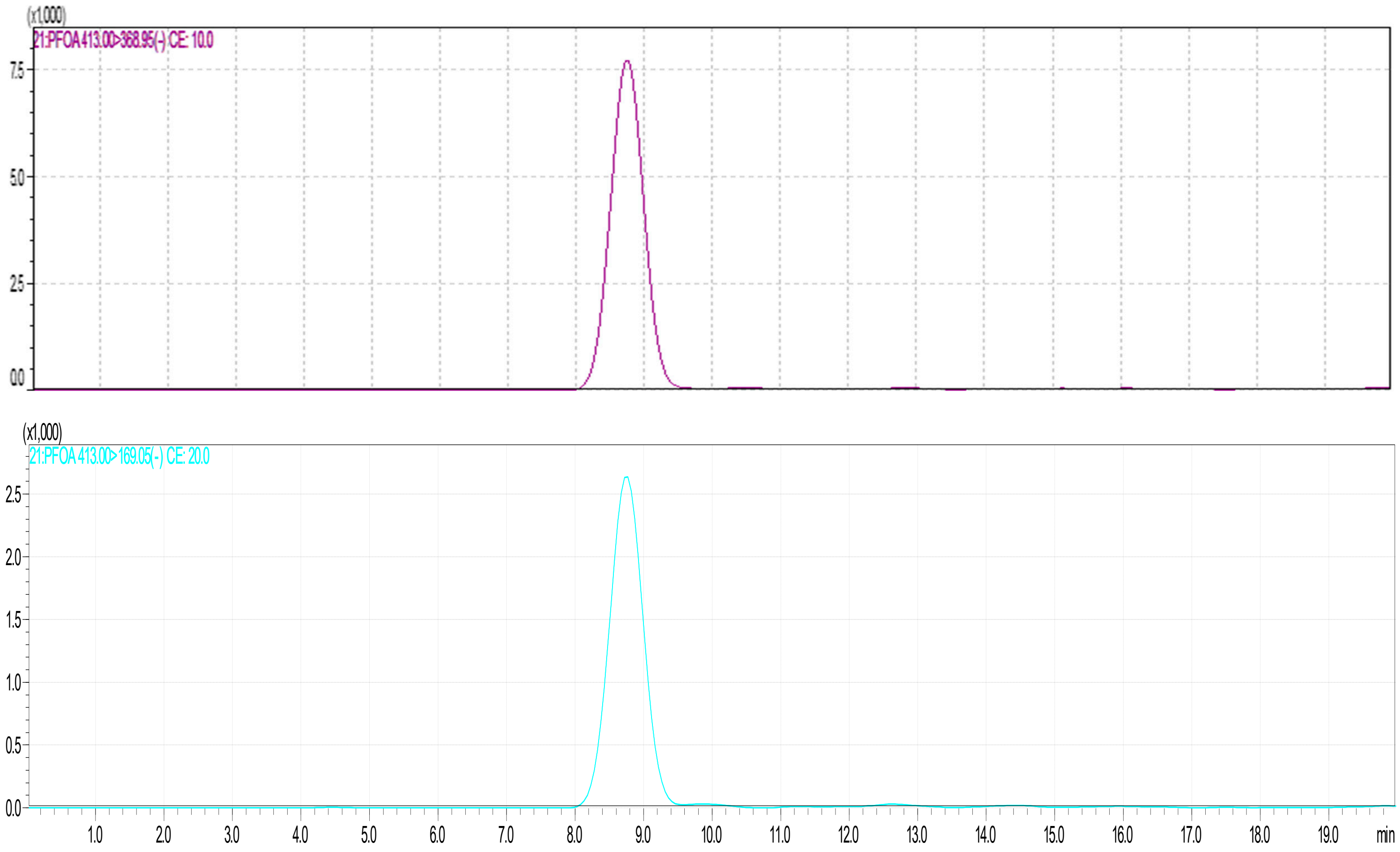

Figure S1. MRM chromatograms of PFBS, PFOS and PFOA.

References

1. Mudumbi, J.B.N.; Ntwampe, S.K.O., Mekuto, L., Matsha, T.; Itoba-Tombo, E.F. The role of pollutants in type 2 diabetes mellitus (T2DM) and their prospective impact on phytomedicinal treatment strategies. *Environ. Monit. Assess.* **2018**, *190*, 262.
2. Rodda, R.; Avvari, S.K.; Chidrawar, R.V.; Reddy, T.R. Pharmacological screening of synergistic antidiabetic efficacy of *Tagetes erecta* and *Foeniculum vulgare*. *Int. J. Phytopharmacol*. **2013**, *4*, 223–229.
3. Hemali, P.;Sumitra, C. Evaluation of antioxidant efficacy of different fractions of *Tagetes erecta* L. Flowers. *J. Pharm. Biol. Sci.* **2014**, *9*, 28–37.
4. Shetty, L.J.; Sakr, F.M.; Al-Obaidy, K.; Patel, M.J.; Shareef, H. A brief review on medicinal plant *Tagetes erecta* Linn A. *J. Appl. Pharm. Sci.* **2015**, *5*, 091–095.
5. Bailung, B.; Puzari, M.. Traditional use of plants by the Ahoms in human health management in upper Assam, India. *J. Med. Plants Stud.* **2016**, *4*, 48–51.
6. Wang, W.; Xu, H.; Chen, H.; Tai, K.; Liu, F.; Gao, Y. In vitro antioxidant, anti-diabetic and antilipemic potentials of quercetagetin extracted from marigold (*Tagetes erecta* L.) inflorescence residues. *J. Food Sci. Technol.* **2016**, *53*, 2614–2624.
7. Davids, D.; Gibson, D.; Johnson, Q. Ethnobotanical survey of medicinal plants used to manage high blood pressure and Type 2 Diabetes mellitus in Bitterfontein, Western Cape Province, South Africa. *J. Ethnopharmacol.* **2016**, *194*, 755–766.
8. Drewes, S.E.; Horn, M.; Khan, F. The chemistry and pharmacology of medicinal plants. In *Commercializing Medicinal Plants—A Southern African Guide*. Drewes, S., Horn, M., Khan, F. Eds.; Sun Press: Stellenbosch: South Aafrica, 2006; pp. 89–95.
9. Van Wyk, B.E.; Albrecht, C. A review of the taxonomy, ethnobotany, chemistry and pharmacology of Sutherlandia frutescens (*Fabaceae*). *J. Ethnopharmacol.* **2008**, *119*, 620–629.
10. Street, R.A.; Prinsloo, G. Commercially important medicinal plants of South Africa: A review. *J. Chem.* **2012**, *2013*, 1–16.
11. Semenya, S.; Potgieter, M.; Erasmus, L. Ethnobotanical survey of medicinal plants used by Bapedi healers to treat diabetes mellitus in the Limpopo Province, South Africa. *J. Ethnopharmacol.* **2012**, *141*, 440–445.
12. Erasto, P.; Adebola, P.O.; Grierson, D.S.; Afolayan, A.J. An ethnobotanical study of plants used for the treatment of diabetes in the Eastern Cape Province, South Africa. *Afr. J. Biotechnol.* **2005**, *4*.
13. Thring, T.S.A.; Weitz, F.M. Medicinal plant use in the Bredasdorp/Elim region of the Southern Overberg in the Western Cape Province of South Africa. *J. Ethnopharmacol.* **2006**, *103*, 261–275.
14. Afolayan, A.J.; Sunmonu, T.O. In vivo studies on antidiabetic plants used in South African herbal medicine. *J. Clin. Biochem. Nutr.* **2010**, *47*, 98–106.
15. van de Venter, M.; Roux, S.; Bungu, L.C.; Louw, J.; Crouch, N.R.; Grace, O.M.; Maharaj, V.; Pillay, P.; Sewnarian, P.; Bhagwandin, N.; et al. Antidiabetic screening and scoring of 11 plants traditionally used in South Africa. *J. Ethnopharmacol.* **2008**, *119*, 81–86.
16. Deutschländer, M.S.; Lall, N.; Van De Venter, M. Plant species used in the treatment of diabetes by South African traditional healers: An inventory. *Pharm. Biol.* **2009**, *47*, 348–365.
17. Loots, D.T.; Pieters, M.; Shahidul Islam, M.; Botes, L. Antidiabetic effects of *Aloe ferox* and *Aloe greatheadii* var. *davyana* leaf gel extracts in a low-dose streptozotocin diabetes rat model. *S. Afr. J. Sci.* **2011**, *107*, 46–51.
18. Balogun, F.O.; Tshabalala, N.T.; Ashafa, A.O.T. Antidiabetic medicinal plants used by the Basotho tribe of Eastern Free State: A review. *J. Diabetes Res.* **2016**, *2016*.
19. Musabayane, C.T.; Xozwa, K.; Ojewole, J.A.O. Effects of *Hypoxis hemerocallidea* (Fisch. & CA Mey.) [Hypoxidaceae] corm (African Potato) aqueous extract on renal electrolyte and fluid handling in the rat. *Ren. Fail.* **2005**, *27*, 763–770.
20. Ojewole, J.A. Antinociceptive, anti-inflammatory and antidiabetic properties of *Hypoxis hemerocallidea Fisch*. & CA Mey. (*Hypoxidaceae*) corm ['African Potato'] aqueous extract in mice and rats. *J. Ethnopharmacol.* **2006**, *103*, 126–134.

21. Gondwe, M.; Kamadyaapa, D.R.; Tufts, M., Chuturgoon, A.A.; Musabayane, C.T. *Sclerocarya birrea* [(A. Rich.) Hochst.] [Anacardiaceae] stem-bark ethanolic extract (SBE) modulates blood glucose, glomerular filtration rate (GFR) and mean arterial blood pressure (MAP) of STZ-induced diabetic rats. *Phytomedicine* **2008**, *15*, 699–709.
22. Oishi, Y.; Sakamoto, T.; Udagawa, H.; Taniguchi, H.; Kobayashi-Hattori, K.; Ozawa, Y.; Takita, T. Inhibition of increases in blood glucose and serum neutral fat by Momordica charantia saponin fraction. *Biosci. Biotechnol. Biochem.* **2007**, *71*, 735–740.
23. Acquaviva, R.; Di Giacomo, C.; Vanella, L.; Santangelo, R.; Sorrenti, V.;Barbagallo, I.; Genovese, C.; Mastrojeni, S.; Ragusa, S.; Iauk, L. Antioxidant activity of extracts of Momordica foetida Schumach. et Thonn. *Mol.* **2013**, *18*, 3241–3249.
24. Sanda, K.A.; Grema, H.A.; Geidam, Y.A.; Bukar-Kolo, Y.M. Pharmacological aspects of Psidium guajava: An update. *Int. J. Pharmacol* **2011**, *7*, 316–324.
25. Mierendorff, H.G.; Stahl-Biskup, E.; Posthumus, M.A.; Beek, T.A.V. Composition of commercial Cape chamomile oil (*Eriocephalus punctulatus*). *Flavour Fragr. J.* **2003**, *18*, 510–514.
26. Njenga, E.W.; Viljoen, A.M. In vitro 5-lipoxygenase inhibition and anti-oxidant activity of Eriocephalus L. (*Asteraceae*) species. *S. Afr. J. Bot.* **2006**, *72*, 637–641.
27. Sandasi, M.; Kamatou, G.P.; Viljoen, A.M. Chemotaxonomic evidence suggests that Eriocephalus tenuifolius is the source of Cape chamomile oil and not Eriocephalus punctulatus. *Biochem. Syst. Ecol.* **2011**, *39*, 328–338.
